# Enhanced electron transportation of PF-NR_2_ cathode interface by gold nanoparticles

**DOI:** 10.1186/s11671-019-3090-z

**Published:** 2019-07-30

**Authors:** Wei Li, Xiaoyan Wu, Guodong Liu, Yanglong Li, Lingyuan Wu, Bo Fu, Weiping Wang, Dayong Zhang, Jianheng Zhao

**Affiliations:** 1 Institute of Fluid Physics, China Academy of Engineering Physics, Mianyang, 621900 China; 2 Key Laboratory of Science and Technology on High Energy Laser, China Academy of Engineering Physics, Mianyang, 621900 China; 3 Institute of Applied Electronics, China Academy of Engineering Physics, Mianyang, 621900 China

**Keywords:** Organic optoelectronic devices, Gold nanoparticle, PF-NR_2_ interface layer, Electron transportation

## Abstract

In order to achieve a wider organic light-emitting diode (OLED) commercial popularity, solution processing inverted polymer light-emitting diode (iPLED) is a trend for further development, but there is still a gap for solution processing devices to achieve commercialization. The improvement of the performance iPLEDs is a research topic of intense current interest. The modification of the cathode interface layer of poly[(9,9-bis(3′-(*N*,*N*-dimethylamino)propyl)-2,7-fluorene)-alt-2,7-(9,9-dioctylfluorene)] (PF-NR_2_) can greatly improve the performance of the devices. However, the electron transportation of the cathode interface layer of PF-NR_2_ films is currently poor, and there is substantial interest in improving its electron transportation to further enhance the performance of organic optoelectronic devices. In this paper, gold nanoparticles (Au NPs) with a particle size of 20 nm were prepared and doped into the interface layer PF-NR_2_ at a specified ratio. The electron transportation of the interface layer of PF-NR_2_ was greatly improved, as judged by conductive atomic force microscopy measurements, which is due to the excellent conductivity of Au NPs. Herein, we demonstrate improved electron transportation of the interface layer by doping Au NPs in PF-NR_2_ film, which provides important and practical theoretical guidance and technical support for the preparation of high performance organic optoelectronic devices.

**Electronic supplementary material:**

The online version of this article (10.1186/s11671-019-3090-z) contains supplementary material, which is available to authorized users.

## Introduction

In the past two decades, organic light-emitting diodes (OLEDs) have attracted wide attention and been studied extensively due to their advantages of flexibility/bendability, diverse material design, easy synthesis and processing, low cost, and light weight. In particular, OLED displays and lighting have begun to realize industrialization and enter the market. Preparation of devices by a solution processing method can reduce the cost and is simple to implement [1–7]. In the past few years, the inverted polymer light-emitting diodes (iPLEDs) have been developed to enhance the stability and rectification ratio. However, there is still a large gap from commercialization for iPLEDs, and the improvement of the performance and life of the devices have become an important topic in current research and depends on the active layer material and interface of the device. In this type of device, charge is directly injected (or extracted) from the electrode to the organic semiconductor layer. Most active layer materials are p-type semiconductors, the number of holes is considerably higher than that of electrons, and high-efficiency devices require carrier injection (or extraction) and transport balance. This requires not only further structural design and modification of the luminescent material, but also methodological improvements in device preparation. Therefore, the properties of the cathode interface layer between the organic active layer and the interface electrode are critical. Therefore, it is necessary to improve the electrical properties of the cathode interface during device preparation [8, 9]. In this type of cathode interface layer, poly[(9,9-bis(3′-(N, N-dimethylamino)propyl)-2,7-fluorene)-alt-2,7-(9,9-dioctylfluorene)] (PF-NR2) is a representative cathode interface modification layer. It has also been previously reported to improve device performance by modifying the PF-NR2 interfacial layer. For example, Huang et al. carried out the addition of an epoxide to PF-NR2 side chains, so that they could undergo a crosslinking reaction on the surface of indium tin oxide (ITO) to enhance the electron transfer. The resulting iPLEDs with the polymer-poly(2-(4-(3′,7′-dimethyloctyloxyphenyl)-1,4-phenylene-vinylene)) (P-PPV) as the light-emitting layer gave a high luminous efficiency of 14.8 cd A−1 [10]. Xie et al. enhanced the electron injection by modifying the PF-NR2 side chain to obtain an all-polymer white light-emitting device with a power efficiency of 11.4 lm W−1 [11]. Chen et al. embedded K+ into the side chains at the interface layer to form a PFCn6:K+ structure, which effectively enhanced the interface conductivity and inhibited the recombination of electron-holes at the interface, so that the power conversion efficiency with poly(3-hexylthiophene): indene-C60 bisadduct (P3HT: ICBA) as the active layer was improved from 5.78 to 7.50% [12]. Generally, the current modifications focusing on the cathode interface layer have all improved the material to enhance its carrier transportation, thereby improving the device performance.

Metal nanoparticles provide photoelectric properties that are available in many materials due to their special volume, quantum size, surface, and macroscopic quantum tunneling effects [13–18]. The performance of the device can be greatly improved by means that include surface-enhanced fluorescence, energy transfer, electrical effects, and scattering effects of metal nanoparticles. Therefore, the application of metal nanoparticles in optoelectronic devices has become a topic of significant interest [19–33]. In this paper, gold nanoparticles (Au NPs) with a particle size of 20 nm were prepared and doped into the interfacial layer of PF-NR2 at a specified ratio. Conductive atomic force microscopy (c-AFM) measurement showed that the electron transportation of the interface layer PF-NR2 was greatly improved. The results indicated that the doping of Au NPs into PF-NR2 could effectively improve the electron transportation of PF-NR2 film, which can be attributed to the excellent conductivity of Au NPs. The Au NPs/PF-NR2 hybrid film was preliminarily introduced into the inverted electroluminescent device, and the enhanced brightness ranged from 17 K cd m−2 to 33 K cd m−2 (94% improvement) and the luminous efficiency was increased from 9.4 cd A−1 to 18.9 cd A−1 (101% improvement). Herein, we investigated PF-NR2 on the surface of Au NPs to improve the electron transportation of the interface layer. The preparation process was simple and efficient, which provides an important and practical theoretical guidance and technical support for the preparation of high-performance iPLEDs.

## Materials and Methods

### Materials

The PF-NR_2_ synthesis process: 2,7-dibromo-9,9-bis(3-(*N*,*N*-dimethylamino)-propyl)fluorene (0.248 g, 0.500 mmol), 2,7-bis(4,4,5,5-tetramethyl-1,3,2-dioxaborolan-2-yl)-9,9-dioctylfluorene (0.321 g, 0.500 mmol), tetrakis (triph-enylphosphine)palladium [(PPh3)4Pd(0)] (10 mg), and several drops of Aliquat 336 were dissolved in a mixture of 3 mL of toluene and 2 mL of 2 M Na_2_CO_3_ aqueous solution. The mixture was refluxed with vigorous stirring for 3 days under an argon atmosphere. After the mixture was cooled to room temperature, it was poured into 200 mL of methanol. The precipitated material was recovered by filtration through a funnel. The resulting solid material was washed for 24 h using acetone to remove oligomers and catalyst residues (0.28 g, 77%).

P-PPV was purchased from Canton OLEDKING Optoelectric Materials Co., Ltd., Guangzhou, China. ITO glass substrates (size 15 × 15 mm ITO) were purchased from China Southern Glass Holding Corp, Shenzhen, China. Poly(3,4-ethylenedioxythiophene):poly(styrene-sulfonate) (PEDOT:PSS, Clevios P AI4083) was bought from Bayer AG.

### Preparation of the Zinc Oxide (ZnO) Precursor

The ZnO precursor was prepared by dissolving zinc acetate dihydrate (Aldrich, 99.9%, 1 g) and ethanolamine (FuYu Fine Chemical Reagent Co., Ltd., 0.28 g) in 2-methoxyethanol (FuYu Fine Chemical Reagent Co., Ltd., 10 mL) under vigorous stirring for 12 h for hydrolysis in air [34, 35].

### Synthesis of Au NPs

The Au NPs used here (20-nm diameter size) were synthesized according to the Frens method [36]. A 100 ml sample of aqueous HAuCl_4_ (0.25 mM, Sinopharm Chemical Reagent Co., Ltd.) was prepared in a 250-ml flask. The solution was brought to boil while being vigorously stirred, with 1 mL of 5% aqueous trisodium citrate dihydrate (Enox) subsequently added. The reaction lasted 15 min until the solution reached a wine red color, indicating the Au NPs of desired size had been synthesized.

### iPLED Device Fabrication

The ZnO precursor solution was spin-coated at 4000 r min^−1^ on top of the ITO-glass substrate. The films were annealed at 200 °C for 1 h in air. The ZnO film thickness was approximately 30 nm. The ZnO-coated substrates were then transferred into a nitrogen-filled glovebox. The PF-NR_2_ interlayer material was dissolved in methanol in the presence of a small amount acetic acid (10 μl ml^−1^), and its solution (concentration = 2 mg ml^−1^) was spin-coated on top of the ZnO film. P-PPV was dissolved into p-xylene with a concentration of 6 and 12 mg mL^−1^, respectively. The P-PPV films were prepared by spin-coating the solution at 1400 r min^−1^ solution onto the buffer layer with a thickness of approximately 80 nm. The pre-devices were then pumped down into vacuum (3 × 10^−4^ Pa). A 10-nm layer of molybdenum oxide (MoO_3_) was thermally deposited on top of the P-PPV layer at an evaporation rate of 0.1 Å s^−1^. Ultimately, a 120-nm Al film was deposited on top of the MoO_3_ layer through a shade mask. The overlap between the cathode and anode defined a 16.0 mm^2^ pixel area. Except for the deposition of the ZnO layers, all other processes were carried out in a controlled atmosphere of nitrogen in a glovebox (Vacuum Atmosphere Co.) containing less than 10 ppm oxygen and moisture.

### Characterization of Devices and Thin Films

#### Conductive Atomic Force Microscopy

The conductivity was tested by Bruker-INNOVA. Conductive atomic force microscopy measurements (Bruker Innova AFM system) were performed in contact mode with a 3 N m^−1^-platinum/iridium-coated silicon cantilever. During the entire scanning process, the setpoint was kept as 1 V. This proper setpoint not only prevented the sample surface from being damaged during the repetitive scanning process, but also ensured the accuracy of the measurement. The local current value was measured by a current amplifier (Femto DLPCA-200) with a current gain of 10^7^ V A^−1^.

Current density–voltage–brightness *(I-V-B*) characteristics were measured in the nitrogen glovebox using a Keithley 236 source measurement unit and a calibrated silicon photodiode. The UV-Vis spectra were recorded by a UV-3600 (SHIMADZU UV-3600). The film thickness was measured by a Dektak 150. The atomic force microscopy (AFM) images were recorded on a Seiko SPA 400 with an SPI 3800 probe station in tapping mode.

## Results and Discussion

### Characterization of Essential Properties of Au NPs and PF-NR_2_ Film

**Fig. 1:**
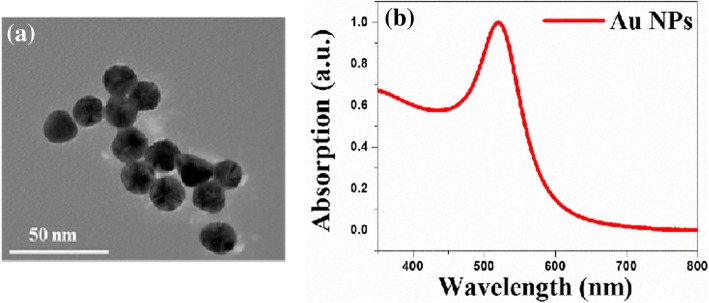
**a** TEM image. **b** Absorption spectra of Au NPs

Au NPs with a particle size of 20 nm (TEM images in Fig. 1a) were prepared by the Frens method and dispersed in an aqueous solution. The absorption spectrum was measured, and its local surface-plasmon resonance (LSPR) peak was found at 520 nm (Fig. 1b). As judged by TEM image and the half peak width of in SPR, the synthesized Au NPs were uniform in size and well dispersed in aqueous solution, which is beneficial to the preparation of the device.

**Fig. 2:**
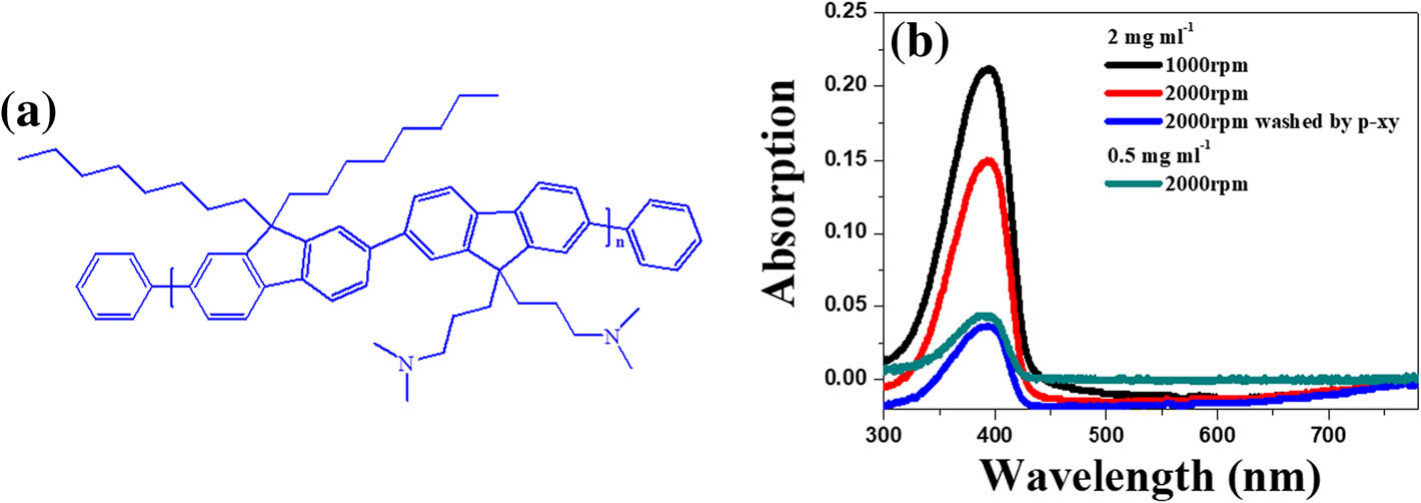
**a** Molecular structure of PF-NR_2_. **b** Thickness variation of PF-NR_2_ under different fabrication conditions measured by UV-Vis spectroscopy

The solution of Au NPs and PF-NR_2_ (chemical structure shown in Fig. 2a) was uniformly mixed at an appropriate ratio (represented by PF-NR_2_/Au NPs), and PF-NR_2_ was prepared by the spin coating method [6]. Because the thickness of the PF-NR_2_ film was too thin at a concentration of 0.5 mg L^−1^ and a speed of 2000 rpm and could not be accurately measured by a surface profilometer, we used a relatively thick PF-NR_2_ film for calibration based on the Lambert-Beer law [10, 37, 38], which states that the absorbance value is proportional to the film thickness (as shown in Fig. 2b). The absorbance value of the PF-NR_2_ film was 0.160 at a concentration of 2 mg L^−1^ and a speed of 1000 rpm, and the film thickness was measured to be 20 nm by surface profilometer. The absorbance value of a PF-NR_2_ film at a concentration of 2 mg L^−1^ and a speed of 2000 rpm washed by p-xy solution was 0.038, and the thickness of the PF-NR_2_ film was calculated to be 5 nm based on the Lambert-Beer law.

**Fig. 3:**
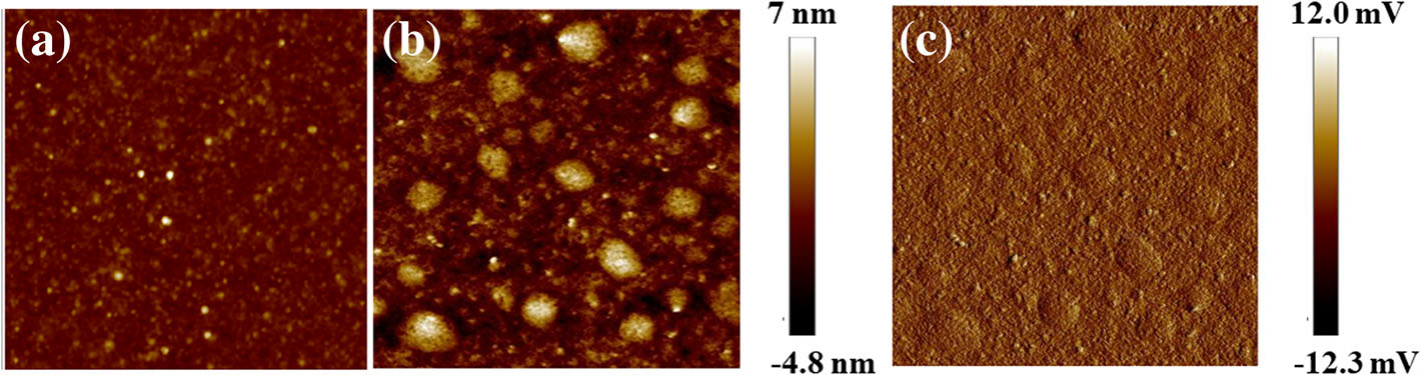
PF-NR_2_ AFM surface morphology **a**, **b** height images without and with Au NPs and **c** phase image with Au NPs (scan area 1.0 μm × 1.0 μm)

Both the PF-NR_2_ film and the PF-NR_2_/Au NP composite film were deposited on an ITO surface. The AFM characterization results of their surface morphologies are shown in Fig. 3a–c. The surface morphology of PF-NR_2_ was changed dramatically after the addition of Au NPs. As the hybrid layer consisted of PF-NR_2_/Au NPs, NPs were clearly observed in the AFM images for the hybrid layer, which showed a root mean square roughness (RMS) increase from 0.562 to 1.590 nm. The interfacial layers both with and without Au NPs are smooth surfaces, allowing high-quality polymer films to be fabricated onto its top. Phase contrast arises from compositional variations of the surface as well as the topographical variations [39]. As seen in Fig. 3c, the phase contrast in PF-NR_2_/Au NPs can be reflected in its topography variation. Apparently, PF-NR_2_/Au NPs shows similar variation tendency in its height and phase images.

### c-AFM Characterization of PF-NR_2_ Thin Films

**Fig. 4:**
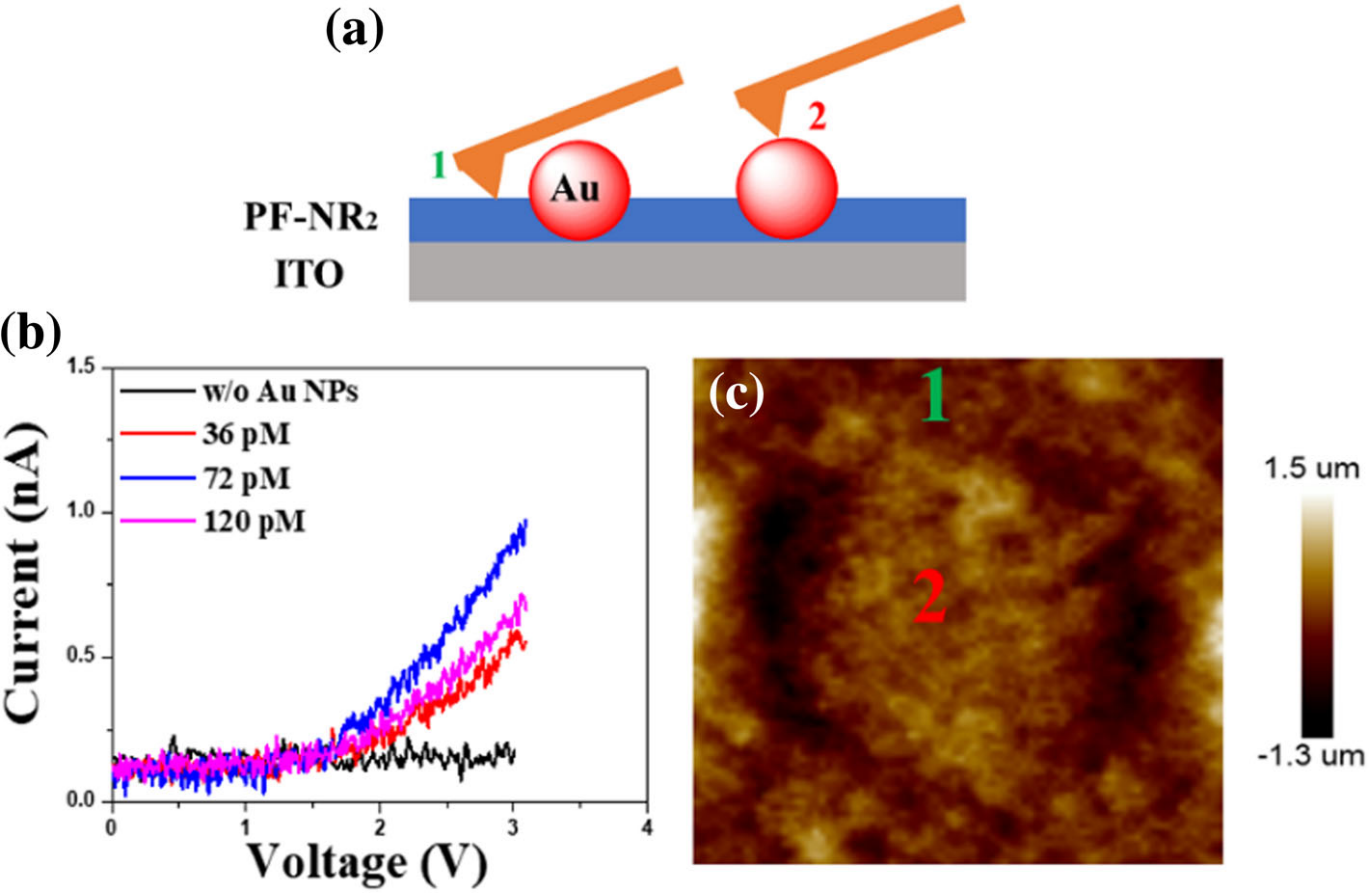
**a** Schematic of c-AFM testing. **b**, **c** I-V characteristics near a single Au NPs and the depiction of the height of a single Au NP in PF-NR_2_ layer. The locations of the colored numbers in the inset image correspond to the color of the I-V curve

**Fig. 5:**
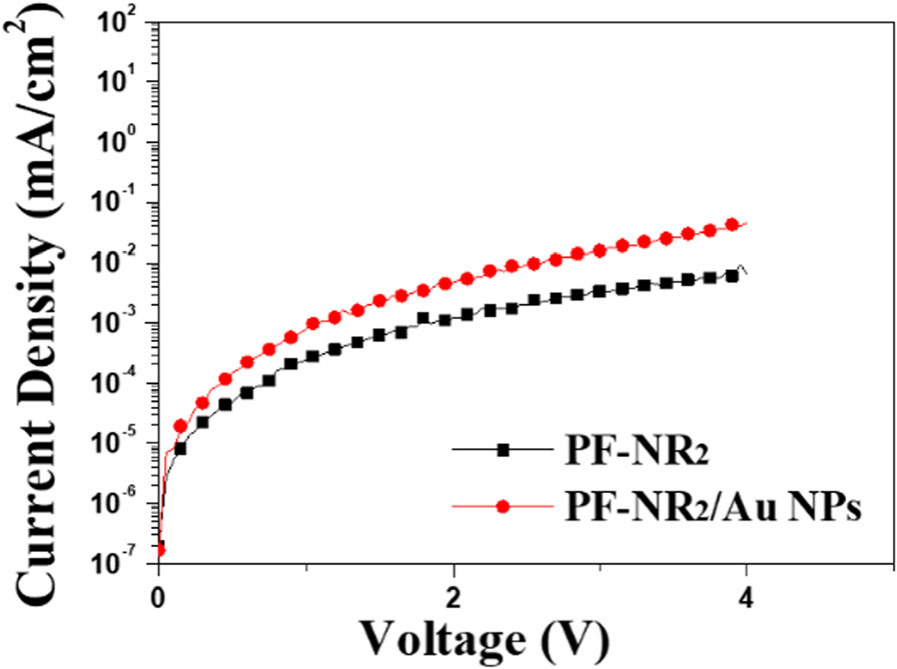
The electron-only devices ITO/ZnO (30 nm)/PF-NR_2_ (5 nm, with and without Au NPs)/P-PPV (80 nm)/CsF (1.5 nm)/Al (120 nm)

**Fig. 6:**
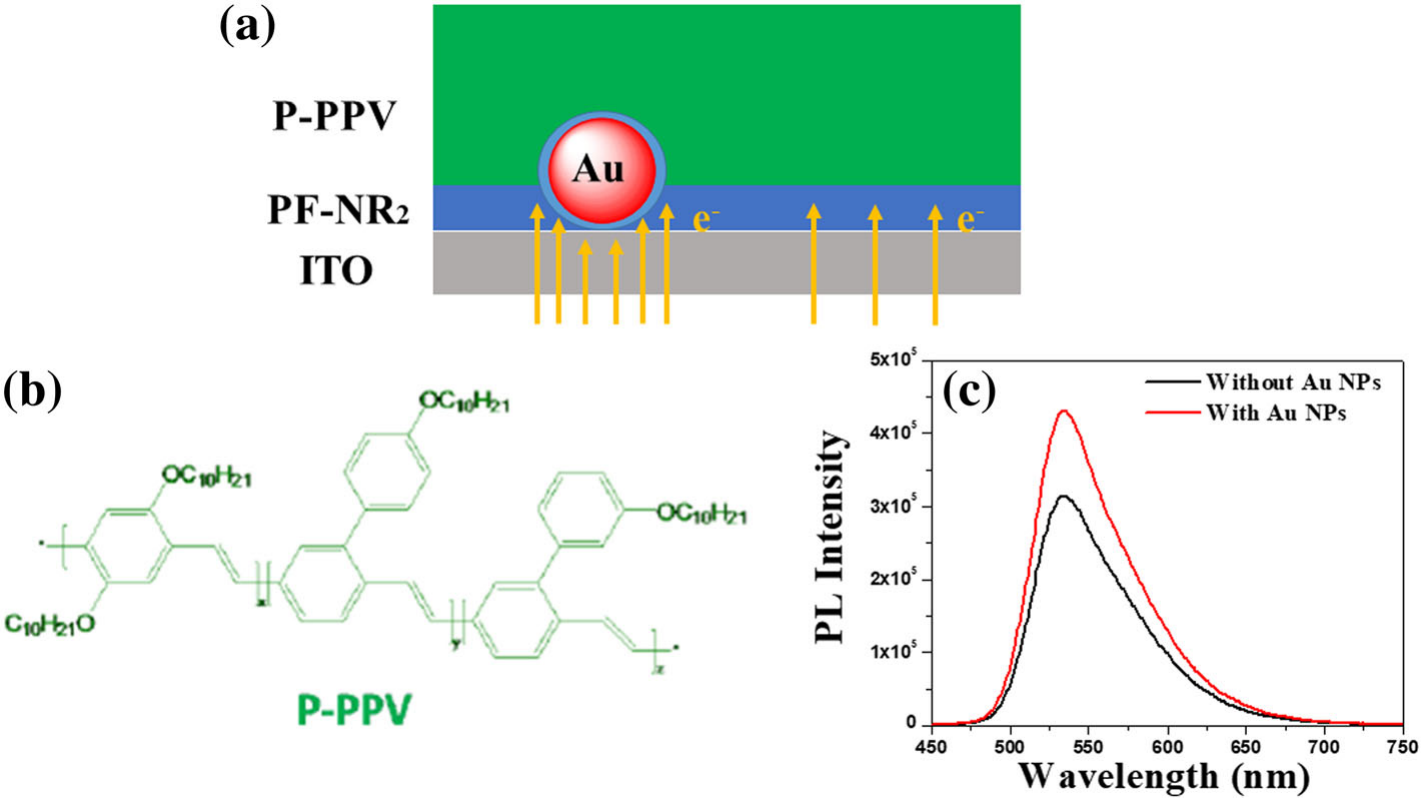
**a** Schematics of the proposed enhanced electron transportation of the hybrid layer with an inverted structure. **b** Molecular structure of PF-NR_2_. **c** PL spectroscopy of P-PPV with and without Au NPs

In order to study the change in the electron transportation of the PF-NR_2_ film after adding Au NPs, we used c-AFM to determine the change in film conductivity. The schematic diagrams of the c-AFM measurements are shown in Fig. 4a–c. We used c-AFM to plot the *I-V* curves of PF-NR_2_/Au NPs with and without Au NPs shown in Fig. 4. At the same time, the electron-only device with the ITO/ZnO (30 nm)/PF-NR_2_ structure (5 nm, with and without Au NPs)/P-PPV (80 nm)/CsF (1.5 nm)/Al (120 nm) has been made to study the effect of Au NPs on electron transportation in Fig. 5. The current increased with the optimized concentration of the Au NPs in Figs. 4b and 5, which indicated that the Au NPs helps in electron injection. Electron transportation of the film with the presence of Au NPs was substantially improved due to the excellent electrical conductivity of the gold nanoparticles. Therefore, the addition of Au NPs to the PF-NR_2_ film can greatly improve the electron transportation of the interface layer. However, when the Au NPs reached a level of 120 pM, the conductivity of the film decreased. The reason might be that an excessively high concentration of Au NPs can cause an aggregation in the PF-NR_2_ film (The SEM image of without, 36 pM, 72 pM, and 120 pM Au NPs doping in PF-NR_2_ has been shown in Additional file 1: Figure S1), and the aggregated Au NPs will considerably reduce the electrical conductivity of the PF-NR_2_ film. We proposed a mechanism for the enhanced conductivity of the device by the Au NPs/PF-NR_2_ thin film, as shown in Fig. 6a. The introduction of Au NPs can improve the electron transportation of the PF-NR_2_ film, thereby enhancing the electron transport capability. Meanwhile, hole-transport is dominant in most polymer luminescent materials, so the improvement of electron transport performance can effectively improve the performance of the device.

In a device structure for general applications, the cathode interface layer will typically be in direct contact with the luminescent layer in iPLEDs. According to Förster energy transfer, if the Au NPs are directly in contact with the luminescent layer, the fluorescence would be quenched. Therefore, we measured the PL spectrum (Fig. 6c) of the luminescent layer based on P-PPV (chemical structure shown in Fig. 6b). As shown from the PL spectral results of the device, the introduction of Au NPs into the PF-NR_2_ film did not quench the fluorescence.

**Fig. 7:**
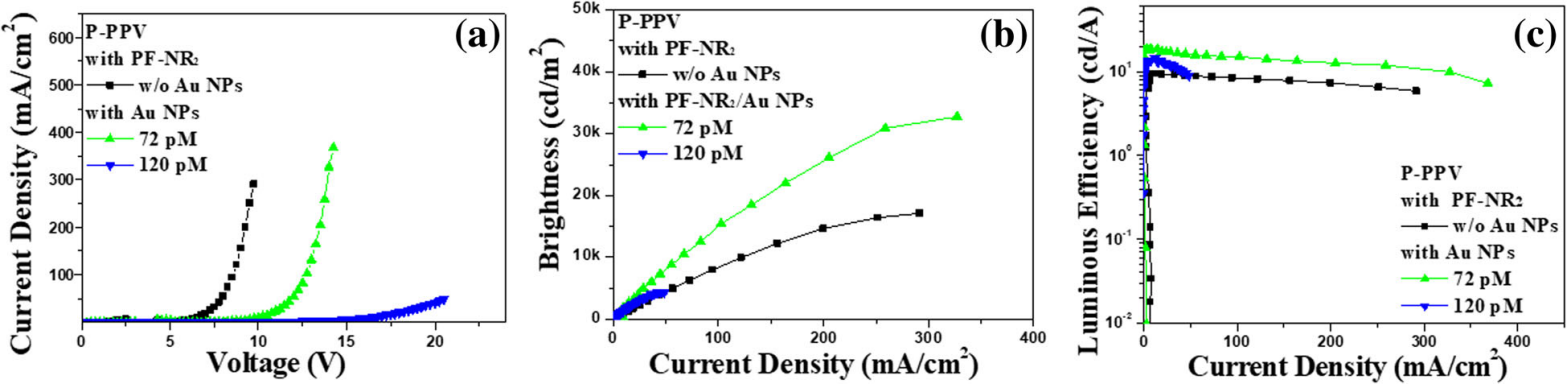
**a** Current density vs. applied voltage (I-V). **b** brightness vs. current density (B-I), and **c** luminous efficiency vs. current density (LE-I) curves under different conditions when P-PPV was used as emitting layer in iPLEDs, respectively

We preliminarily applied the PF-NR_2_/Au NP composite film to the iPLEDs with a device structure of ITO/ZnO (30 nm)/PF-NR_2_ (5 nm, with or without Au NPs)/P-PPV (80 nm)/MoO_3_ (10 nm)/Al (120 nm), the enhanced brightness ranged from 17 K cd m^−2^ to 33 K cd m^−2^ (94% improvement), and the luminous efficiency was increased from 9.4 cd A^−1^ to 18.9 cd A^−1^ (101% improvement), as shown in Fig. 7a–c. Based on our previous research conclusion, the weak improvement of PL intensity made little contribution to the device performance [19, 25]. The significant improvement in device performance indicates that Au NPs can improve the electron transportation of PF-NR_2_ and improve electron transport efficiency, thus enhancing the electron-hole recombination efficiency. With comprehensive consideration device efficiency, AFM phase imaging, and PL spectra, we conclude that the PF-NR_2_ film partially adhered to the surface of the Au NPs, which avoided direct contact of Au NPs with the luminescent layer P-PPV [40].

## Conclusions

In this study, we prepared Au NPs with a size of about 20 nm by the Frens method, and the Au NPs were doped into the interface layer PF-NR_2_ at a specified ratio. It was found that the electron transportation of the interface layer PF-NR_2_ was effectively improved due to the excellent conductivity of Au NPs, while the interface layer of PF-NR_2_/Au NPs did not quench the fluorescence emission of the luminescent layer. Because most of the luminescent materials in devices are p-type semiconductors, the number of holes is substantially higher than that of electrons, and high-efficiency devices require carrier injection and transport balance. Therefore, improving the electron transportation of the cathode interface layer is a key method to effectively increase the efficiency of the device. Herein, an effective way to improve the electron transportation of the interface layer PF-NR_2_ by an Au NP interface doping was proposed, and the preparation process was simple and effective, which is important for preparing high-efficiency iPLEDs.

## Additional file

Additional file 1: **Figure S1.** The SEM images of without, 36 pM, 72 pM, and 120 pM Au NP doping in PF-NR_2_. (DOCX 393 kb)

## Data Availability

The datasets used and/or analyzed during the current study are available from the corresponding author on reasonable request.

## Abbreviations

AFM: Atomic force microscopy
Au NPs: Gold nanoparticles
B-I: Brightness vs. current density
c-AFM: Conductive atomic force microscopy
iPLED: Inverted polymer light-emitting diode
ITO: Indium tin oxide
I-V: Current density vs. applied voltage
I-V-B: Current density–voltage–brightness
LE-I: Luminous efficiency vs. current density
LSPR: Local surface plasmon resonance
OLED: Organic light-emitting diode
P3HT: ICBA: Poly(3-hexylthiophene): indene-C60 bisadduct
PEDOT:PSS: Poly (3,4-ethylenedioxythiophene):poly(styrene-sulfonate)
PF-NR_2_: Poly[(9,9-bis(3′-(*N*,*N*-dimethylamino)propyl)-2,7-fluorene)-alt-2,7-(9,9-dioctylfluorene)]
PL: Photoluminescence
PLED: Polymer light-emitting diode
P-PPV: Polymer-poly(2-(4-(3′,7′-dimethyloctyloxyphenyl)-1,4-phenylene-vinylene))
TEM: Transmission electron microscope
