# Supplementary material for: Enhanced electron transportation of PF-NR2 cathode interface by gold nanoparticles
Source: Nanoscale Res Lett. 2019 Jul 30;14:261. doi: 10.1186/s11671-019-3090-z (PMC6667568; doi:10.1186/s11671-019-3090-z)
Supplement: Supplementary file 1 — Figure S1. The SEM images of without, 36 pM, 72 pM, and 120 pM Au NP doping in PF-NR2. (DOCX 393 kb) [file 11671_2019_3090_MOESM1_ESM.docx]

Additional file

Enhanced electron transportation of PF-NR_2_ cathode interface by gold nanoparticles

Wei Li ^1,2^, Xiaoyan Wu ^1,,2,^*, Guodong Liu^1,2,^*,Yanglong Li^1,2^, Lingyuan Wu^1,2^, Bo Fu^1,2^, Weiping Wang^1,2^, Dayong Zhang^1^, Jianheng Zhao^3^

^1^ Institute of Fluid Physics, China Academy of Engineering Physics, Mianyang 621900, China. [vleefoxtrot@outlook.com](mailto:vleefoxtrot@qq.com) (W. L.); [wuxiaoyan1219@sina.cn](mailto:wuxiaoyan1219@sina.cn) (X. W.); [guodliu@126.com](mailto:guodliu@126.com) (G. L.); [jesseliyl@163.com](mailto:jesseliyl@163.com) (Y. L.); [wubaly@163.com](mailto:wubaly@163.com) (L. W.); [fubo_phoenix@163.com](mailto:fubo_phoenix@163.com) (B. F.); [wwpwzc@yeah.net](mailto:wwpwzc@yeah.net) (W. W.); [zdywxl874@sohu.com](mailto:zdywxl874@sohu.com) (D. Z.); [jianh_zhao@caep.cn](mailto:jianh_zhao@caep.cn) (J. Z.).

^2^ Key Laboratory of Science and Technology on High Energy Laser, China Academy of Engineering Physics, Mianyang 621900, China. [vleefoxtrot@outlook.com](mailto:vleefoxtrot@qq.com) (W. L.); [wuxiaoyan1219@sina.cn](mailto:wuxiaoyan1219@sina.cn) (X. W.); [guodliu@126.com](mailto:guodliu@126.com) (G. L.); [jesseliyl@163.com](mailto:jesseliyl@163.com) (Y. L.); [wubaly@163.com](mailto:wubaly@163.com) (L. W.); [fubo_phoenix@163.com](mailto:fubo_phoenix@163.com) (B. F.); [wwpwzc@yeah.net](mailto:wwpwzc@yeah.net) (W. W.) ; [zdywxl874@sohu.com](mailto:zdywxl874@sohu.com) (D. Z.).

^3^ Institute of Applied Electronics, China Academy of Engineering Physics, Mianyang 621900, China. [jianh_zhao@caep.cn](mailto:jianh_zhao@caep.cn) (J. Z.).

***** Correspondence: [wuxiaoyan1219@sina.cn](mailto:wuxiaoyan1219@sina.cn), [guodliu@126.com](mailto:guodliu@126.com); Tel.: +86-0816-2487145


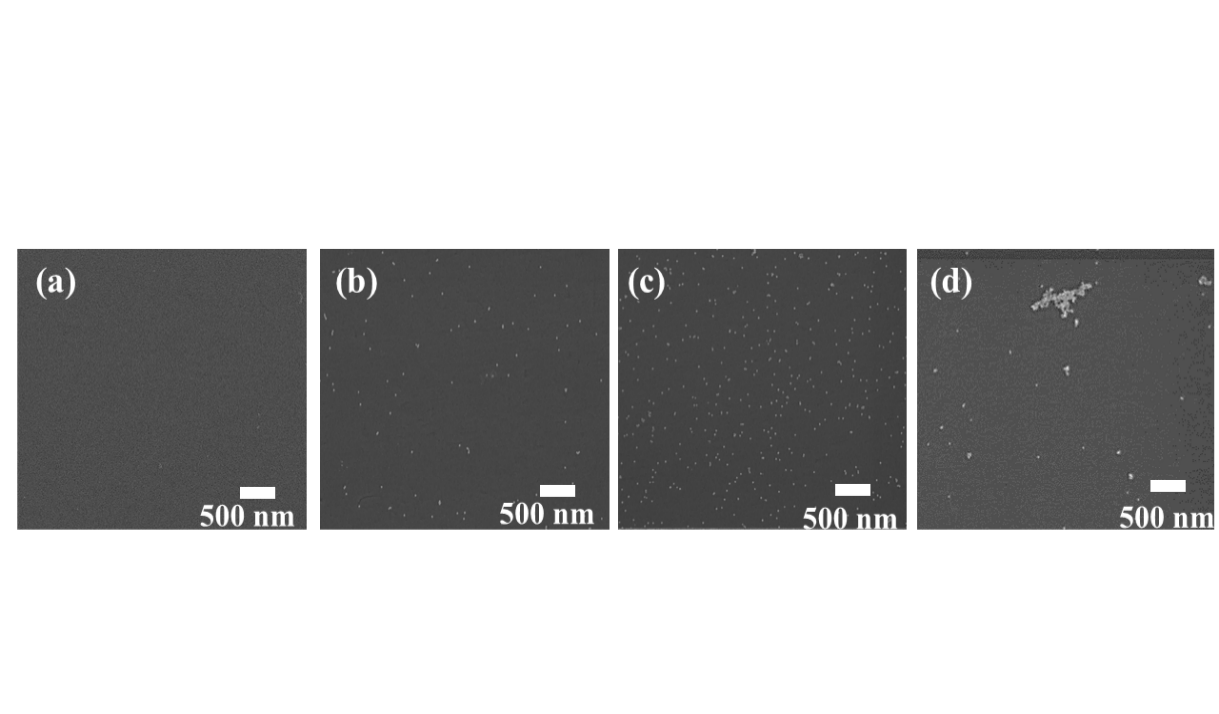


**Figure S1**. The SEM images of without, 36 pM, 72 pM and 120 pM Au NPs doping in PF-NR_2_.
